# Blood Metabolomics May Discriminate a Sub-Group of Patients with First Demyelinating Episode in the Context of RRMS with Increased Disability and MRI Characteristics Indicative of Poor Prognosis

**DOI:** 10.3390/ijms232314578

**Published:** 2022-11-23

**Authors:** Marina Boziki, Alexandros Pechlivanis, Christina Virgiliou, Christos Bakirtzis, Styliani Aggeliki Sintila, Eleni Karafoulidou, Evangelia Kesidou, Paschalis Theotokis, Ioannis Nikolaidis, Georgios Theodoridis, Helen Gika, Nikolaos Grigoriadis

**Affiliations:** 1 Laboratory of Experimental Neurology and Neuroimmunology and Multiple Sclerosis Center, 2nd Neurological University Department, AHEPA General Hospital of Thessaloniki, Aristotle University of Thessaloniki, 54636 Thessaloniki, Greece; 2 Biomic_AUTh, Center for Interdisciplinary Research and Innovation (CIRI-AUTH), Balkan Center, 57001 Thessaloniki, Greece; 3 Laboratory of Analytical Chemistry, Department of Chemistry, Aristotle University of Thessaloniki, 54124 Thessaloniki, Greece; 4 Laboratory of Forensic Medicince and Toxicology, School of Medicine, Aristotle University of Thessaloniki, 54124 Thessaloniki, Greece

**Keywords:** metabolomics, first demyelinating episode, Relapsing-Remitting Multiple Sclerosis, Clinically Isolated Syndrome, biomarkers, disability, prognosis

## Abstract

Biomarker research across the health-to-disease continuum is being increasingly applied. We applied blood-based metabolomics in order to identify patient clusters with a first demyelinating episode, and explored the prognostic potential of the method by thoroughly characterizing each cluster in terms of clinical, laboratory and MRI markers of established prognostic potential for Multiple Sclerosis (MS). Recruitment consisted of 11 patients with Clinically Isolated Syndrome (CIS), 37 patients with a first demyelinating episode in the context of Relapsing-Remitting MS (RRMS) and 11 control participants. Blood-based metabolomics and hierarchical clustering analysis (HCL) were applied. Constructed OPLS-DA models illustrated a discrimination between patients with CIS and the controls (*p* = 0.0014), as well as between patients with RRMS and the controls (*p* = 1 × 10^−5^). Hierarchical clustering analysis (HCL) for patients with RRMS identified three clusters. RRMS-patients-cluster-3 exhibited higher mean cell numbers in the Cerebro-spinal Fluid (CSF) compared to patients with CIS (18.17 ± 6.3 vs. 1.09 ± 0.41, *p* = 0.004). Mean glucose CSF/serum ratio and infratentorial lesion burden significantly differed across CIS- and HCL-derived RRMS-patient clusters (F = 14.95, *p* < 0.001 and F = 6.087, *p* = 0.002, respectively), mainly due to increased mean values for patients with RRMS-cluster-3. HCL discriminated a cluster of patients with a first demyelinating episode in the context of RRMS with increased disability, laboratory findings linked with increased pathology burden and MRI markers of poor prognosis.

## 1. Introduction

Over the last decade, advances in high-throughput techniques have made it possible to study the protein and metabolic profile in relation to genetic and phenotypic diversity in several multifactorial diseases, including neurodegenerative diseases [1,2]. These techniques have particular application in Multiple Sclerosis (MS), a disease of the Central Nervous System (CNS) with a neurodegenerative component and with high heterogeneity regarding the clinical course and the degree of response to disease-modifying treatments (DMTs) [3,4].

Clinically Isolated Syndrome (CIS) [5] has been associated with an increased likelihood of conversion to MS [6,7,8,9,10]. The identification of biomarkers that timely indicate increased likelihood of transition from CIS to MS has been the subject of extensive research. Moreover, patients with a first demyelinating episode in the context of Relapsing-Remitting MS (RRMS) are also a highly heterogenous group in terms of clinical presentation, MRI characteristics and prognosis. The identification of prognostic biomarkers in patients newly diagnosed with RRMS has been recognized as a target of particular significance [10,11]. A number of CNS-derived metabolites have been identified and pathogenetically linked with MS [12,13,14]. Few studies have addressed serum metabolomics in CIS versus RRMS, as well as across the disease trajectory [12,15,16,17], thus highlighting the potential of the method to provide valuable information regarding disease pathology and evolution [18].

The aim of the present study is to apply blood metabolomic analysis in order to identify distinct serum metabolomic patterns among patients with different phenotypic characteristics in the context of a first demyelinating episode that fulfills either CIS or RRMS diagnosis. Notably, most existing studies in the field address the potential of metabolomics to discriminate between overall different conditions or disease forms; for instance, in a case—control setting or RRMS vs. secondary progressive MS (SPMS). These studies explore the diagnostic potential of metabolomics but do not address issues of prognosis. In the context of the present study, we apply detailed phenotyping with respect to attributes, both clinical and MRI, that have been linked with differential prognostic value. By thoroughly dissecting, in terms of phenotype, the heterogeneity that characterizes patients newly diagnosed with RRMS, we aim to link differential metabolomic profiles to patient clusters that exhibit laboratory and/or radiological attributes with prognostic potential.

## 2. Results

### 2.1. Study Population

Recruitment consisted of 11 patients with CIS, 37 patients with a first demyelinating episode in the context of RRMS and 11 control participants. Participant characteristics are presented in Table 1. One patient in the CIS Group exhibited increased IgG index, accounting for unusually increased mean IgG index for the CIS Group; however, this patient did not fulfill dissemination in space criterion for the fulfillment of RRMS diagnosis and, therefore, remained in the CIS Group. All participants were within the normal weight range and followed a standard diet.

### 2.2. Metabolomic Analysis May Discriminate between Patients with Cis and Healthy Controls, as Well as between Patients with a First Demyelinating Episode in the Context of RRMS and Healthy Controls

**Figure 1:**
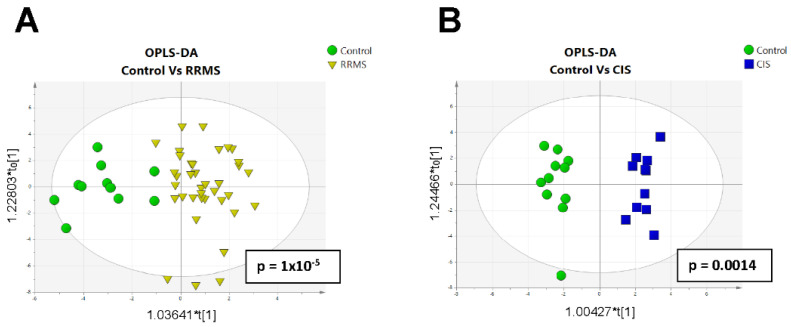
Orthogonal Projection to Latent Structures Discriminant Analysis (OPLS-DA) of (**A**) RRMS vs. controls; (**B**) CIS vs. controls. CIS: Clinically Isolated Syndrome; RRMS: Relapsing-Remitting Multiple Sclerosis.

In total, 46 metabolites were detected in blood samples, including amines, amino acids, organic acids, carbohydrates, purines and other polar metabolites (Supplementary Table S1). Unbiased PCA analysis was performed in order to assess intrabatch precision. QC samples were clustered together indicating analytical system stability (Supplementary Figure S1). Further analysis by OPLS DA was performed between the studied groups. Constructed OPLS-DA models illustrated a clear discrimination between patients with CIS and the controls (*p* = 0.0014), as well as between patients with RRMS and the controls (*p* = 1 × 10^−5^) (Figure 1A,B).

Metabolomic analysis was not able to discriminate between CIS and RRMS. Permutation tests showed that the models were robust, with high predictability (R2Y(cum) and Q2 values of 0.758 and 0.547 for (RRMS vs. Control), respectively, and 0.799 and 0.479 for CIS vs. Control). Cross-validation ANOVA testing (CV-ANOVA) was significant for the two OPLS models (*p* < 0.05). The constructed multivariate models, in combination with the univariate statistical analysis, enabled highly significant features to be revealed.

**Figure 2:**
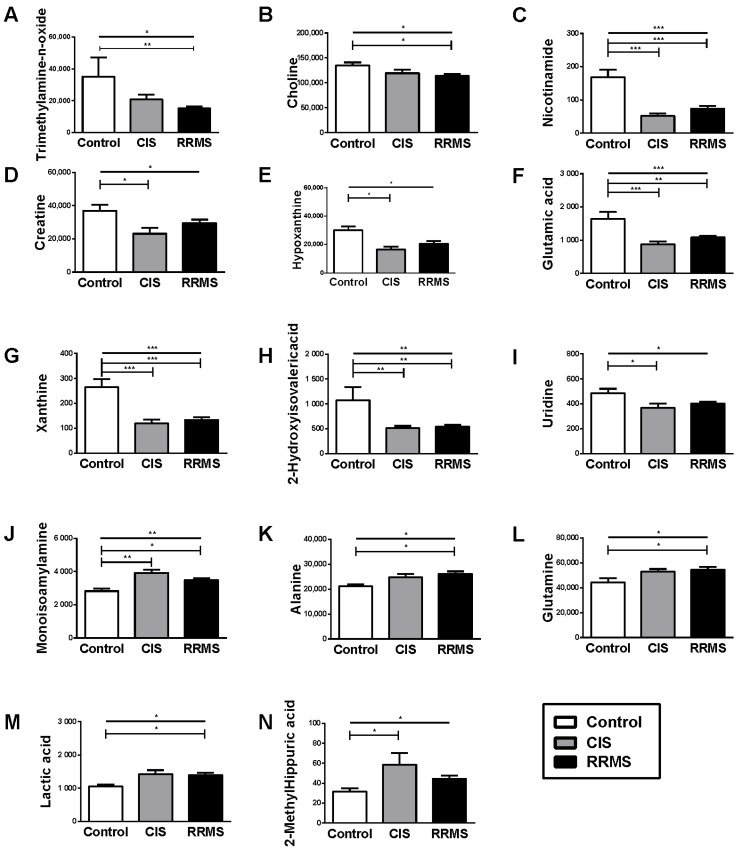
Variation of statistically significant metabolomics’ compounds in each of the studied group. (**A**) trimethylamine-n-oxide; (**B**) choline; (**C**) nicotinamide; (**D**) creatine; (**E**) hypoxanthine; (**F**) glutamic acid; (**G**) xanthine; (**H**) 2-Hydroxyisovalericacid; (**I**) uridine; (**J**) monoisoamylamine; (**K**) alanine; (**L**) glutamine; (**M**) lactic acid; (**N**) 2-MethylHippuric acid. Bars ± error bars indicate mean ± standard error of mean. Line with open edges indicates overall comparison among the three groups. Lines with vertical edges indicate post-hoc comparisons. CIS: Clinically Isolated Syndrome; RRMS: Relapsing-Remitting Multiple Sclerosis. * *p* < 0.05; ** *p* < 0.01; *** *p* < 0.001.

Metabolomic analysis exhibited pronounced alterations in the signal of 15 metabolites in the blood of patients with RRMS and 15 metabolites in the blood of patients with CIS, compared to the controls. Among the statistically significant compounds, nine metabolites were found to differentiate between RRMS vs. the controls, as well as between CIS vs. the controls. Specifically, monoisoamylamine, amino acids including alanine, glutamine and the organic acid lactate, were increased in RRMS vs. the controls, as well as in CIS vs. the controls. Conversely, nicotinamide, xanthine and glutamic acid were decreased in RRMS vs. the controls, as well as in CIS vs. the controls, showing especially strong impact; as were uridine and hypoxanthine. Essential amino acids tryptophan and methionine together with aspartic acid, serine and 2-methylhippuric acid were shown to mostly differentiate patients with CIS from either patients with RRMS or the controls. Choline, pyruvic acid, creatine, trimethylamine-n-oxide and 2–hydroxy isovaleric acid were shown to mostly differentiate patients with RRMS from either CIS or the controls (Table 2 and Figure 2).

**Figure 3:**
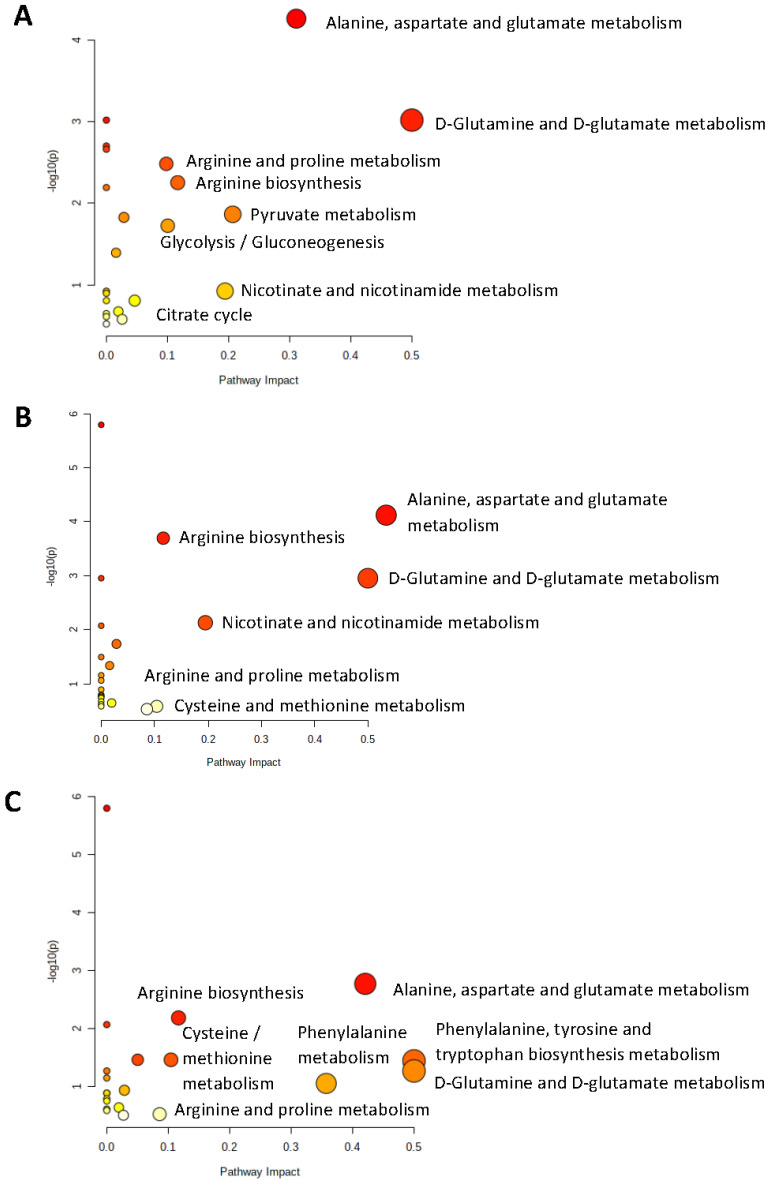
Highly influenced biochemical pathways, as indicated by significant metabolites that contribute to group differentiation: (**A**) Relapsing-Remitting Multiple Sclerosis (all patients) vs. control, (**B**) Clinically Isolated Syndrome vs. control, (**C**) Relapsing-Remitting Multiple Sclerosis (clusters 2 3) vs. Clinically Isolated Syndrome. Metabolome view presents all matched pathways according to the *p* values derived from the pathway enrichment analysis and pathway impact values derived from the pathway topology analysis. Graphs were derived from the online web software MetaboAnalyst 5.0. Pathways that strongly contribute to group differentiation are depicted in bigger and reddish colored cycle. RRMS: Relapsing-Remitting Multiple Sclerosis; CIS: Clinically Isolated Syndrome.

The metabolic pathways identified to be perturbated between patients with RRMS vs. the controls and between patients with CIS vs. the controls are shown in Figure 3A,B.

### 2.3. Exploratory Hierarchical Clustering Analysis for Patients with RRMS Reveals Three Sub-Populations, One with a Common Metabolomic Profile with CIS

**Figure 4:**
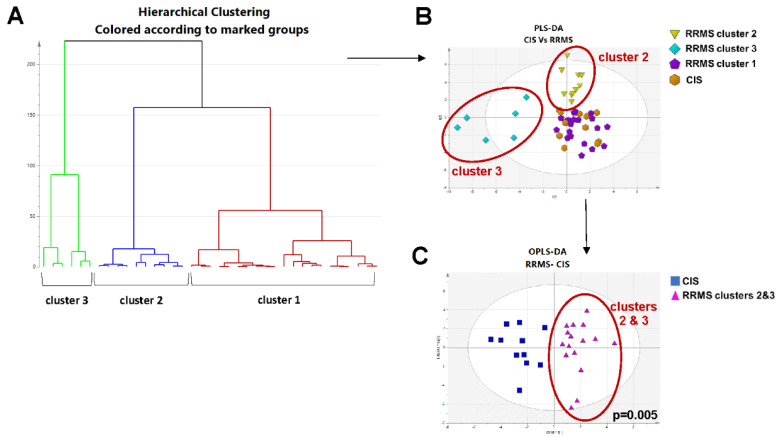
(**A**) Hierarchical Clustering Analysis dendrogram of samples from Relapsing-Remitting Multiple Sclerosis group using the original data and parameters from multivariate analysis (Principal Components Analysis model, 3 components: cluster 1 in red, overlapping with Clinically Isolated Syndrome, cluster 2 in blue and cluster 3 in green, UV scaling), (**B**) Projection to Latent Structures Discriminant Analysis (PLS-DA); observations (samples) are colored according to cluster from Hierarchical Clustering Analysis and symbol for cases with according to classes, where triangles in yellow represent cases with Clinically Isolated Syndrome, (**C**) Orthogonal Projection to Latent Structures Discriminant Analysis of Clinically Isolated Syndrome against Relapsing-Remitting Multiple Sclerosis clusters 2 and 3, following exclusion of Relapsing-Remitting Multiple Sclerosis cluster 1, that is, Relapsing-Remitting Multiple Sclerosis cluster overlapping to Clinically Isolated Syndrome. HCA: Hierarchical Clustering Analysis; PLS-DA: Projection to Latent Structures Discriminant Analysis; OPLS-DA: Orthogonal Projection to Latent Structures Discriminant Analysis; RRMS: Relapsing-Remitting Multiple Sclerosis; CIS: Clinically Isolated Syndrome. PLS-DA was used as a mean to visualize samples and clusters after hierarchical clustering analysis. OPLS-DA was chosen for visualization of classification between the cluster 2 and 3 of RRMS samples and CIS samples.

Hierarchical clustering analysis was further performed in order to explore potential spontaneous clustering of patients with RRMS, based on their metabolomic profile. Three main clusters were identified based on the generated dendrogram from a “bottom-up” HCA of the RRMS group using data/parameters from the respective multivariate model (PCA-X analysis) (Figure 4A). The three clusters (*n* = 6, *n* = 10, *n* = 21) of RRMS samples (green, blue and red nodes) were further assessed against CIS samples over a new PLS-DA. Of the three RRMS clusters, cluster 1 overlaps with samples from the CIS group, whereas clusters 2 and 3 appear as distinct populations (Figure 4B). This was demonstrated in a separate OPLS-DA score plot that included the CIS patients and only patients with RRMS from clusters 2 and 3, according to which the model was able to accurately discriminate between patients with CIS vs. RRMS cluster 2 and 3 patients (*p* = 0.005) (Figure 4C). The model provided a statistically significant differentiation between the case and control groups. The model’s validity was cross-verified by R2Y and Q2Y values, which were found to be 0.813 and 0.476, respectively, as well as by the model’s CV-ANOVA value (*p* < 0.05).

With respect to compounds responsible for the differentiation between RRMS clusters 2 and 3 and CIS, univariate analysis revealed 14 possible markers. Among them, hypoxanthine, xanthine, monoisoamylamine and glutamic acid were also found to be significant when diseased groups were separately assessed against controls. Betaine, cysteine, monoisoamylamine and trimethylamine n-oxide (TMAO) were observed at increased concentrations in the blood of patients diagnosed with CIS compared to RRMS, while the opposite was observed for the amino acids phenylalanine, serine, methionine, aspartic acid, asparagine, glutamic acid and threonine, purines including xanthine and hypoxanthine and the organic acid 2-hydroxyisobutyric acid (Table 2). The underlying biochemical traits may involve a modified amino acid metabolism that may directly or indirectly link to the disease (Figure 3C).

### 2.4. Metabolomics’ Hierarchical Clustering Analysis Discriminates a Sub-Population of Patients with RRMS with Increased Disability upon the First Demyelinating Episode, Laboratory Findings Suggestive of Increased Neuroinflammation and MRI Markers of Poor Prognosis

When CIS and RRMS clusters 1–3 were considered, mean EDSS differed between the four groups (F = 3.103, *p* = 0.036, Table 3). Upon post-hoc comparisons, there was a tendency for a significant difference between patients with CIS and patients with RRMS cluster 2 (0.95 ± 0.21 vs. 2.5 ± 0.48, respectively, *p* = 0.067), whereas the other mean EDSS post-hoc comparisons did not reach statistical significance. However, patients with CIS evidently exhibited lower mean EDSS compared to patients with RRMS clusters 1–3 (Table 3), though this difference did not reach statistical significance. Moreover, patients with RRMS cluster 1, in addition to exhibiting a mostly overlapping serum metabolomic profile to patients with CIS, also exhibited lower mean EDSS compared to patients with RRMS clusters 2–3, though this difference did not reach statistical significance. Notably, CIS corresponds, at least in part, to a clinical entity that frequently indicates prodromal MS. Patients with RRMS cluster 1 evidently exhibited a partial proximity to CIS patients in terms of serum metabolic profile and lower mean disability scores, compared to patients with RRMS clusters 2 and 3. Based on these observations, we subsequently tested the hypothesis that metabolomic-derived HCL for patients with a first demyelinating episode in the context of RRMS may indicate populations with distinct clinical attributes that may differ in terms of severity, and these populations may also exhibit laboratory and/or MRI attributes indicative of a more advanced underlying pathology and/or poor prognosis.

Mean ALP, TSH and ESR differed across patient groups, considering patients with CIS and patients with RRMS clusters 1–3 (F = 3.372, *p* = 0.028; F = 3.783, *p* = 0.018 and F = 2.765, *p* = 0.054, respectively; Table 3).

However, in post-hoc comparisons, no statistical difference was reached. Mean cell numbers in the CSF significantly differed across patient groups (F = 4.789, *p* = 0.006) and this difference was mainly attributed to patients with RRMS cluster 3, who exhibited higher mean cell numbers in the CSF compared to patients with CIS (18.17 ± 6.3 vs. 1.09 ± 0.41, *p* = 0.004) and a tendency for higher mean cell numbers in the CSF to patients with RRMS cluster 1 (18.17 ± 6.3 vs. 7.1 ± 2.1, *p* = 0.071). Glucose in the CSF significantly differed across patient groups (F = 27.772, *p* < 0.001) and this difference was exclusively attributed to patients with RRMS cluster 3, who exhibited higher mean glucose in the CSF compared to patients with CIS (95.67 ± 4.18 vs. 64.09 ± 1.36, *p* < 0.001), to patients with RRMS cluster 1 (95.67 ± 4.18 vs. 63.43 ± 1.13, *p* < 0.001) and to patients with RRMS cluster 2 (95.67 ± 4.18 vs. 65 ± 4.51, *p* < 0.001). As suggested in clinical practice, we also calculated the CSF/serum glucose ratio, based on simultaneous measurements [19]. Glucose CSF/serum ratio significantly differed across patient groups (F = 14.95, *p* < 0.001) and this difference was exclusively attributed to patients with RRMS cluster 3 who exhibited higher mean CSF/serum ratio compared to patients with CIS (1.1 ± 0.07 vs. 0.76 ± 0.03, *p* < 0.001), to patients with RRMS cluster 1 (1.1 ± 0.07 vs. 0.71 ± 0.02, *p* < 0.001) and to patients with RRMS cluster 2 (1.1 ± 0.07 vs. 0.77 ± 0.07, *p* < 0.001). IgG SCF to serum ratio significantly differed across groups (F = 3.523, *p* = 0.023) and this difference was attributed to patients with CIS who exhibited lower IgG CSF to serum ratio compared to patients with RRMS cluster 1 (0.64 ± 0.04 vs. 1.05 ± 0.14, *p* = 0.017). However, as the revised diagnostic criteria for RRMS includes evidence of intrathecal IgG synthesis, where necessary, inclusion of CIS in the overall comparison poses an inherent bias due to the fact that these patients, almost always by definition, do not show evidence of intrathecal IgG synthesis. When the analysis was conducted only for RRMS patient clusters 1–3, the groups did not differ in terms of IgG SCF to serum ratio (F = 1.132, *p* = 0.334).

**Figure 5:**
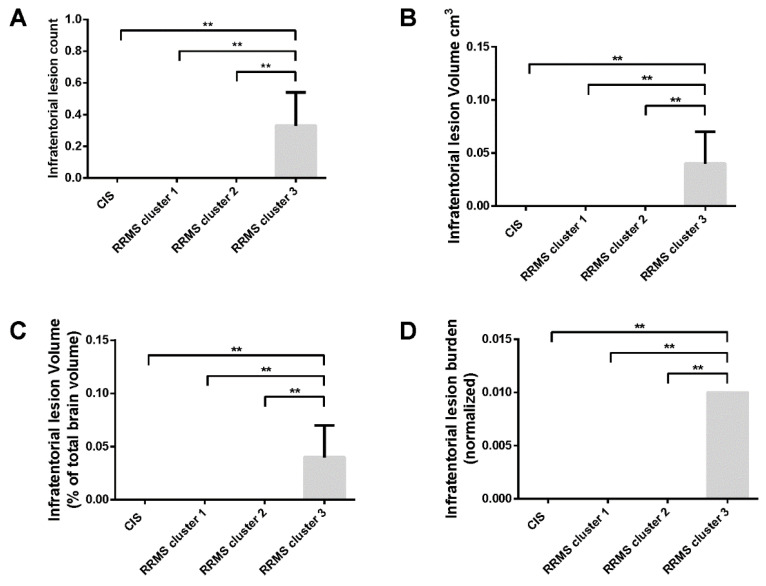
Magnetic Resonance Imaging lesion analysis with respect to infratentorial lesion parameters for patients with Clinically Isolated Syndrome and Relapsing-Remitting Multiple Sclerosis stratified into three clusters (1–3) derived from Hierarchical Cluster Analysis on the basis of serum metabolomic profile. CIS: Clinically Isolated Syndrome; RRMS: Relapsing-Remitting Multiple Sclerosis. Brackets indicate post-hoc comparisons. Bars and error bars represent mean ± standard error of mean. ** *p* < 0.01.

Mean infratentorial lesion count, mean infratentorial lesion volume (absolute; cm^3^), mean infratentorial normalized lesion volume and mean infratentorial lesion burden differed across groups, considering patients with CIS and patients with RRMS clusters 1–3 (F = 6.087, *p* = 0.002; F = 6.087, *p* = 0.002; F = 6.087, *p* = 0.002 and F = 6.087, *p* = 0.002, respectively; Supplementary Table S2). Following post-hoc comparisons with respect to all four measurements of infratentorial lesion analysis, the overall difference was attributed to the increased mean value exhibited by patients with RRMS cluster 3, compared to patients with CIS and to patients with RRMS clusters 1 and 2 (Supplementary Table S2 and Figure 5).

The patient groups did not differ with respect to other volumetry and/or lesion analysis parameters on brain MRI (Supplementary Table S2). Although patients with RRMS cluster 3 exhibited a higher mean number of spinal T2 lesions compared to patients with CIS and patients with RRMS clusters 1 and 2, the difference did not reach statistical significance (2.7 ± 1.02 vs. 0.6 ± 0.16, 1.8 ± 0.45 and 2 ± 0.53, respectively, F = 2.069, *p* = 0.12; Table 3). When the mean number of infratentorial and spinal T2 lesions in total was compared across patient groups, a tendency for a significant difference was evident (F = 2.587, *p* = 0.068; Table 3), attributed to a tendency for increased mean number of lesions in patients with RRMS cluster 3, compared to patients with CIS (3 ± 0.86 vs. 0.56 ± 0.18, *p* = 0.057).

### 2.5. Distinct Serum Metabolomic Profile in Patients with RRMS Cluster 3, Compared to Patients with RRMS Clusters 1 and 2

**Figure 6:**
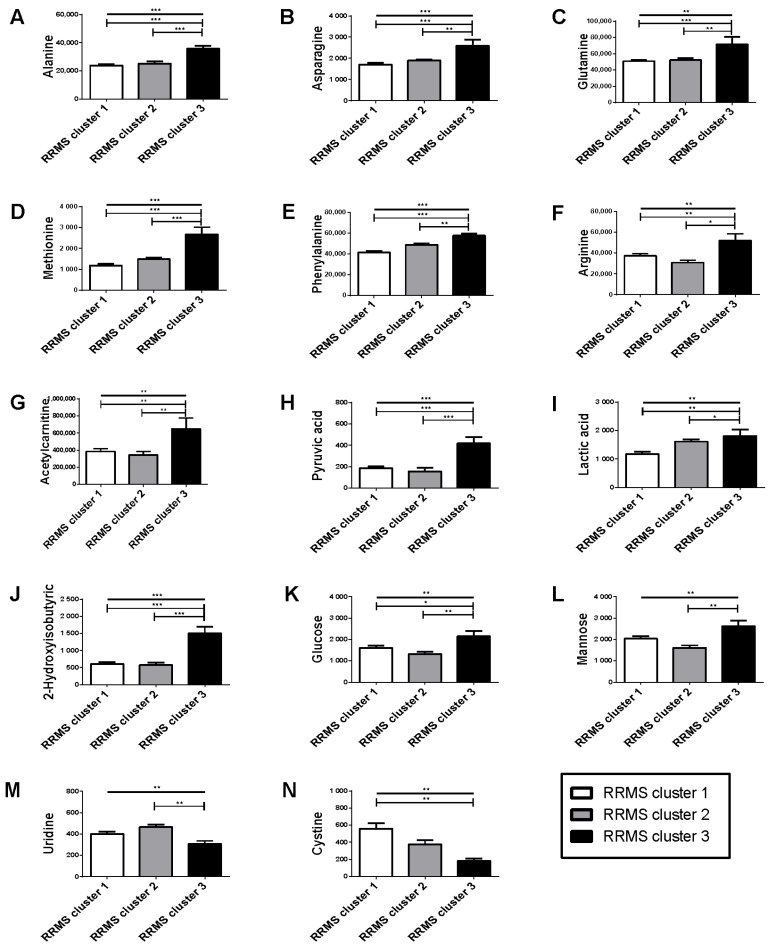
Variation of statistically significant metabolites in each of the studied clusters of cases with Relapsing-Remitting Multiple Sclerosis. (**A**) alanine; (**B**) asparagine; (**C**) glutamine; (**D**) methionine; (**E**) phenylalanine; (**F**) arginine; (**G**) acetylcarnitine; (**H**) pyruvic acid; (**I**) lactic acid; (**J**) 2-Hydroxyisobutyric; (**K**) glucose; (**L**) mannose; (**M**) uridine; (**N**) cystine. Bars ± error bars indicate mean ± standard error of mean. Line with open edges indicates overall comparison among the three groups. Lines with vertical edges indicate post-hoc comparisons. * *p* < 0.05; ** *p* < 0.01; *** *p* < 0.001.

A total of 11 serum metabolites, namely alanine, asparagine, glutamine, methionine, phenylalanine, arginine, acetylcarnitine, cystine, pyruvic acid, lactic acid and 2-hydroxybutyric acid exhibited increased concentrations in patients with RRMS cluster 3 compared to patients with RRMS clusters 1 and 2, thus underlining an, at least in part, distinct serum metabolomic profile in patients with RRMS cluster 3 compared to the other patients with RRMS. Serum glucose was evidently increased in patients with RRMS cluster 3 compared to patients with RRMS cluster 2, whereas it was comparable between clusters 3 and 1. Similarly, mannose and uridine exhibited reduced serum concentration in patients with RRMS cluster 3, but this difference was observed either in comparison to patients with RRMS cluster 2 or to patients with RRMS cluster 1, respectively; thus not signifying a pattern distinct for patients with RRMS cluster 3 (Table 4 and Figure 6).

## 3. Discussion

In the present study, blood-based metabolomics were able to accurately classify between patients with first demyelinating episode in the context of RRMS and the controls, as well as between patients with CIS and the controls, but not between patients with CIS and patients with first demyelinating episode in the context of RRMS. To our knowledge, this is the first study that addresses blood-based metabolomic profiles for patients with CIS, sub-populations of patients with a first demyelinating episode in the context of RRMS, and for healthy controls. The identification of patients with CIS at increased risk of potentially transitioning to RRMS is currently made on the basis of MRI and few clinical characteristics [20,21]. Similarly, the degree of further disease activity in patients with a first demyelinating episode in the context of RRMS remains mostly unpredictable. The observation that blood metabolomic analysis may discriminate between healthy controls and patients with a neuroinflammatory disease, either CIS or first demyelinating episode in the context of RRMS, supports the potential of the approach in elucidating markers of neuroinflammatory disease in the peripheral blood.

Exploratory hierarchical clustering analysis for patients with a first demyelinating episode in the context of RRMS revealed three sub-populations, one with a common metabolomic profile with CIS (cluster 1). Moreover, the same approach was able to discriminate a sub-population of patients with a first demyelinating episode in the context of RRMS (cluster 3), who exhibit increased disability upon the demyelinating episode, distinct laboratory findings, though consistent with MS diagnosis, and MRI markers linked with poor disease prognosis.

More specifically, patients with a first demyelinating episode in the context of RRMS (cluster 3) exhibited higher mean disability scores compared to patients with a first demyelinating episode in the context of RRMS (clusters 1 and 2), and to patients with CIS. Interestingly, patients with RRMS cluster 3 exhibited higher mean cell numbers in the CSF compared to patients with CIS, and a tendency for higher mean cell numbers in the CSF to patients with RRMS cluster 1; thus showing, at least in part, evidence of increased CNS neuroinflammation [22]. Blood–brain barrier (BBB) damage has been well described in the Relapsing-Remitting, mostly inflammatory stage of CNS autoimmune demyelination, and has been associated with increased disease severity [23].

Moreover, patients with RRMS cluster 3 exhibited higher mean CSF glucose, as well as glucose CSF/serum ratio, compared to patients with CIS, patients with RRMS cluster 1 and patients with RRMS cluster 2. Energetic defects in neurons, however, are a well-described attribute of chronic pathology in the frame of MS [24]. Neural cultures treated with CSF from patients with progressive MS were shown to exhibit signs of mitochondrial dysfunction leading to increased expression of glucose transporter molecules, in an attempt to increase glucose uptake from the environment, thus compensating the energetic defect [25]. Similarly, upregulation of the glucose transporter system has also been described in acute MS lesions in an autopsy-based study [26]. The upregulation effect was partly rescued in vitro by glucose supplementation of the environment [25]; an observation that may, at least in part, explain increased glucose CSF/serum ratio in patients with RRMS cluster 3, who exhibit an overall increased severity of the first demyelinating episode.

With respect to all four measurements of infratentorial lesion analysis, patients with RRMS cluster 3 exhibited increased mean values compared to patients with CIS and patients with RRMS clusters 1 and 2. Furthermore, in our study, patients with RRMS cluster 3 exhibited a higher mean number of spinal T2 lesions compared to patients with CIS, to patients with RRMS cluster 1 and to patients with RRMS cluster 2, although the difference did not reach statistical significance. Infratentorial T2 lesions have been associated with poor long-term prognosis for patients with a first demyelinating episode, and infratentorial lesion burden has been advocated as a radiological marker for the identification of patients with MS at high risk for earlier disability accumulation [27]. Furthermore, the early presence of spinal cord T2 lesions has been similarly linked with poor prognosis in patients with CIS and/or RRMS [28,29]. Taken together, our results indicate that patients with RRMS cluster 3 show MRI evidence of poor prognosis with a higher likelihood of early disability accumulation, compared to patients with CIS and to patients with RRMS clusters 1 and 2.

Metabolomics provided evidence of pathways implicated in disease pathogenesis. Alanine-aspartate and glutamine/glutamate metabolism were pathways that exhibited major impacts on the differentiation between RRMS patients and the controls, as well as between CIS patients and the controls, thus signifying important metabolic alterations in the transition from health towards CNS disease. Metabolic alterations have been described in the context of the diseased CNS, as a response to increased energy needs derived from defects in neuron energy production [30,31]. Glutamate in particular has been previously identified as an energy source for neuronal and non-neuronal CNS cell populations in the context of disease, and has been implicated in Reactive Oxygen Species generation and in pathways of oxidative stress [32,33]. In MS, recent evidence regarding mitochondria dysfunction and oxidative stress-related pathology has underlined the presence of neurodegenerative components early in the disease course [34,35,36]. In this respect, metabolomic pathway analysis may assist in the identification of patients with a first demyelinating episode that exhibit evidence of an increased neurodegenerative pathological component, a pathology linked with poor long-term clinical outcomes.

Patients with RRMS clusters 2 and 3 exhibited additional alterations in energy metabolism (pyruvate metabolism, TCA cycle, glycolysis/gluconeogenesis) compared to CIS patients, such as phenylalanine, tyrosine and tryptophan biosynthesis. This observation is in line with recent reports that serum metabolomics identifies alterations in serum aromatic amino acid (AAA) metabolites in MS, linked with disease severity [37,38]. Moreover, patients with RRMS cluster 3 exhibited increased plasma arginine and methionine concentrations, the latter being implicated in oxidative stress and its regulation [39], and previously correlated with MRI markers of pathology in patients with RRMS [40]. Our results are further supported by recent evidence providing proof-of-principle data regarding the potential of blood-based metabolomics to elucidate pathways of pathogenesis in MS. Notably, four metabolic pathways, namely glycerophospholipid, citrate cycle, sphingolipid, and pyruvate metabolism, have been identified to be altered in RRMS compared to control subjects, with the glycolysis pathway being a common upstream metabolic pathway. These results were coupled with experimental evidence stemming from mice with Experimental Autoimmune Encephalomyelitis, in which modulation of glycolysis resulted in disease amelioration with a profound anti-inflammatory effect in the innate immunity system of the CNS [41].

Our study does have limitations. First, participant numbers differed across study groups, due to limited recruitment of control and CIS patients. The control group poses inherent limitations in terms of recruitment rates as, overall, few hospitalized patients remain free from a neurological pathology. Moreover, according to the latest revision of the diagnostic criteria for MS [8], few patients with a first demyelinating episode remain under CIS diagnosis. Second, possible heterogeneity of patients with CIS with respect to their risk of transition towards RRMS was not addressed, as this would require a prospective setting. However, the quality of symptoms at onset, describing the severity of the first demyelinating episode, has been a well-described factor of short-term prognosis in patients with early MS [42,43,44]. In this respect, special effort was made towards thorough phenotyping of the participants. Notably, brain lesion quantification analysis was derived from VolBrain^™^ [45], a tool that, to our knowledge, shows inherent limitations in discriminating between lesions with or without typical MS-like morphology; a limitation that may, at least in part, account for increased brain lesion numbers in the CIS group. For this reason, all MRI studies were evaluated by the treating neurologists of the Center, as well as one treating neurologist who independently evaluated all MRI studies for reasons of internal consistency and internal quality control. Third, in the frame of the present study, CSF samples were not analyzed for metabolomics. CSF-based metabolomic analysis is expected to be more direct, depicting the condition of the CNS in a more accurate manner. In this respect, CSF-based metabolomic analysis may potentially contribute as a validation method for the observed metabolomic alterations in blood, a method lacking from the present study. However, blood-based biomarkers are a significant research target of increased value, relative to the CSF-based biomarkers, due to the restricted access in the CSF and the fact that lumbar puncture is indicated primarily upon the diagnostic evaluation of a demyelinating disease with few indications afterwards over the course of the disease. In the frame of the present study, clinical and MRI biomarkers with known prognostic value were used in order to link them with blood-based metabolomic alterations. Moreover, as comprehensive lipids are important metabolites for the function of CNS, untargeted metabolomics or lipidomics, in addition to the targeted 100 metabolites explored in the frame of the present study, are expected to provide more abundant information in the hunt of biomarkers.

## 4. Materials and Methods

All participants were recruited at the Multiple Sclerosis (MS) Center of the 2nd University Neurological Clinic of A.U.TH. in the University General Hospital of Thessaloniki AHEPA, following written informed consent. The study was conducted in accordance with the Declaration of Helsinki and approved by the Research Ethics and Conduct Committee of the A.U.TH. (AEDE AUTH) [Approval Nr. 112730/2021]. Methods are reported in Supplementary Material’s “Supplementary Material and Methods” citing references [8,45,46,47,48,49].

## 5. Conclusions

To our knowledge, this is the first study to apply blood-based metabolomic analysis in order to identify distinct serum metabolomic patterns among patients with different phenotypic characteristics in the context of a first demyelinating episode that fulfills either CIS or RRMS diagnosis. The present study provides evidence that blood-based metabolomic analysis applied in patients with a first demyelinating episode in the context of RRMS may identify a patient population characterized by increased clinical severity at onset, distinct laboratory characteristics suggestive of the underlying pathology and MRI markers linked with poor long-term clinical outcomes. As prognosis upon the first demyelinating episode is limited and is mainly based on clinical and/or MRI attributes with limited potential for clinical practice, the identification of additional markers of prognostic potential is of significant value for CIS/MS management. Such aspects of disease management include therapeutic decisions with respect to DMT administration in the CIS, the optimal DMT choice for treatment initiation in MS and the overall treatment plan with respect to DMT escalation and the timely initiation of high efficacious DMTs, in the framework of internationally applied treatment guidelines.

## Figures and Tables

**Table 1:** Demographic, clinical, laboratory and radiological characteristics of participants.

	CIS		RRMS		Control		
	Mean	S.E.	Mean	S.E.	Mean	S.E.	*p* *
age	38.55	1.72	36.32	2.03	33.45	2.06	0.531
gender (m/f)	6/5	N/A	14/23	N/A	6/5	N/A	0.458
**EDSS**	**0.95**	**0.21**	**2.27**	**0.24**	N/A	N/A	**0.006**
VitD	20.06	2.6	21.21	1.59	N/A	N/A	0.715
B12	335.88	58.81	347.05	29.1	N/A	N/A	0.865
Folate	20.2	4.97	16.23	1.92	N/A	N/A	0.389
TSH	1.41	0.29	2.03	0.19	N/A	N/A	0.125
**IgG CSF/IgG serum ×1000**	**3.47**	**0.37**	**5.71**	**0.42**	N/A	N/A	**<0.001**
**IgG INDEX**	**0.64**	**0.04**	**0.96**	**0.08**	N/A	N/A	**0.001**
brain T2W lesions **	21.2	3.27	17.94	1.48	N/A	N/A	0.328
**brain Gd(+) lesions**	**0**	**0**	**1.14**	**0.3**	N/A	N/A	**<0.001**
**spinal T2W lesions**	**0.6**	**0.16**	**2**	**0.33**	N/A	N/A	**0.001**
**spinal Gd(+) lesions**	**0**	**0**	**0.52**	**0.18**	N/A	N/A	**0.007**

CIS: Clinically Isolated Syndrome; RRMS: Relapsing-Remitting Multiple Sclerosis; S.E.: Standard Error of Mean; m: male; f: female; EDSS: Expanded Disability Status Scale; VitD: Vitamin D; B12: Vitamine B12; TSH: Thyroid Stimulating Hormone; CSF: cerebro-spinal fluid; IgG: immunoglobulin class G; Gd(+): gadolinium-enhancing; N/A: non-applicable. * One-Way Analysis of Variance / Independent Student’s *t*-test/Pearson’s Chi-square, where applicable. ** data derived from VolBrain^TM^ lesion analysis. Bold denotes comparisons with *p* < 0.05.

**Table 2:** Metabolites implicated in the differentiation between the studied groups.

Compounds	F	RRMS (All Patients) vs. Control			CIS vs. Control			CIS vs. RRMS (RRMS: Clusters 2 and 3)		
		*t*-Test	AUC	log2 FC	*t*-Test	AUC	log2 FC	*t*-Test	AUC	log2 FC
2- Methylhippuric acid	0.031	>0.05	N/A	NA	3.26 × 10^−2^	0.79	0.88		N/A	N/A
Alanine	0.036	9.63 × 10^−3^	0.78	0.32	3.92 × 10^−2^	0.75	0.21	>0.05	N/A	N/A
Asparagine	0.334	>0.05	N/A	NA	>0.05	N/A	N/A	6.97 × 10^−3^	0.69	−0.19
Aspartic acid	0.278	>0.05	N/A	NA	2.60 × 10^−2^	0.78	−0.52	2.68 × 10^−3^	0.54	−0.30
Betaine	0.785	>0.05	N/A	NA	>0.05	N/A	N/A	8.78 × 10^−3^	0.64	0.18
Choline	0.025	9.06 × 10^−3^	0.75	−0.23	>0.05	N/A	N/A	>0.05	N/A	N/A
Creatine	0.042	4.37 × 10^−2^	0.73	−0.38	>0.05	N/A	N/A	>0.05	N/A	N/A
Cystine	0.861	>0.05	N/A	NA	>0.05	N/A	N/A	3.74 × 10^−3^	0.61	0.12
Glutamic acid	<0.001	5.34 × 10^−4^	0.78	−0.60	2.30 × 10^−3^	0.88	−0.88	4.13 × 10^−2^	0.63	−0.28
Glutamine	0.045	2.24 × 10^−2^	0.68	0.30	4.28 × 10^−2^	0.70	0.24	>0.05	N/A	N/A
Hydroxyisobutyric acid	0.217	>0.05	N/A	NA	>0.05	N/A	N/A	3.78 × 10^−2^	0.66	−0.47
Hydroxyisovaleric acid	0.001	9.56 × 10^−4^	0.73	−1.02	>0.05	N/A	N/A	>0.05	N/A	N/A
Hypoxanthine	0.02	3.04 × 10^−2^	0.83	−0.55	2.74 × 10^−4^	0.91	−0.91	1.79 × 10^−2^	0.59	−0.37
Lactic acid	0.034	1.25 × 10^−2^	0.76	0.43	6.71 × 10^−3^	0.81	0.43	>0.05	N/A	N/A
Methionine	0.261	>0.05	N/A	NA	4.88 × 10^−2^	0.73	−0.32	2.44 × 10^−3^	0.70	−0.42
Monoisoamylamine	0.001	5.86 × 10^−3^	0.77	0.32	4.24 × 10^−4^	0.90	0.45	3.58 × 10^−2^	0.62	0.13
Nicotinamide	<0.001	2.03 × 10^−5^	0.86	−1.19	4.83 × 10^−5^	0.91	−1.73	>0.05	N/A	N/A
Phenylalanine	0.341	>0.05	N/A	NA	>0.05	N/A	N/A	1.21 × 10^−3^	0.70	−0.14
Pyruvic acid	0.109	4.66 × 10^−2^	0.71	0.70	>0.05	N/A	N/A	>0.05	N/A	N/A
Serine	0.074	>0.05	N/A	N/A	2.94 × 10^−2^	0.71	−0.26	1.20 × 10^−3^	0.66	−0.21
Threonine	0.217	>0.05	N/A	N/A	2.18 × 10^−2^	0.76	−0.32	2.94 × 10^−2^	0.71	−0.37
Trimethylamine-n-oxide	0.011	4.92 × 10^−3^	0.68	−1.24	>0.05	N/A	N/A	2.25 × 10^−2^	0.70	0.48
Uridine	0.029	2.07 × 10^−2^	0.68	−0.27	3.76 × 10^−2^	0.73	−0.35	>0.05	N/A	N/A
Xanthine	<0.001	1.19 × 10^−5^	0.86	−0.97	3.12 × 10^−4^	0.92	−1.21	2.50 × 10^−2^	0.61	−0.24

Only statistically significant metabolites in at least one comparison together with the results from univariate analysis, including *t*-test, fold change analysis and area under the curve, are summarized. RRMS: Relapsing-Remitting Multiple Sclerosis; CIS: Clinically Isolated Syndrome; Log2 FC: logarithmic 2-fold change; AUC: area under the curve; N/A: non-available.

**Table 3:** Clinical, radiological and laboratory parameters for patients with Clinically Isolated Syndrome and for patients with Relapsing-Remitting Multiple Sclerosis stratified into three clusters (1–3) derived from Hierarchical Cluster Analysis on the basis of serum metabolomic profile.

Parameter/HCL Group		*n*	Mean	S.E. of Mean	F *	p *
EDSS	CIS	11	0.95	0.21	3.103	0.036
	RRMS cluster 1	21	2.07	0.33		
	RRMS cluster 2	10	2.50	0.48		
	RRMS cluster 3	6	2.58	0.49		
ALP	CIS	9	56.33	3.21	3.372	0.028
	RRMS cluster 1	16	65.44	3.50		
	RRMS cluster 2	10	72.70	4.47		
	RRMS cluster 3	6	56.17	4.77		
TSH	CIS	9	1.41	0.29	3.783	0.018
	RRMS cluster 1	18	2.49	0.28		
	RRMS cluster 2	10	1.51	0.23		
	RRMS cluster 3	6	1.51	0.30		
SER 60 min	CIS	9	6.78	1.18	2.765	0.054
	RRMS cluster 1	21	17.14	2.81		
	RRMS cluster 2	10	10.60	1.73		
	RRMS cluster 3	5	11.40	2.98		
cells (CSF)	CIS	11	1.09	0.41	4.789	0.006
	RRMS cluster 1	21	7.10	2.10		
	RRMS cluster 2	10	9.90	2.59		
	RRMS cluster 3	6	18.17	6.30		
glucose (CSF)	CIS	11	64.09	1.36	27.772	<0.001
	RRMS cluster 1	21	63.43	1.13		
	RRMS cluster 2	9	65.00	4.51		
	RRMS cluster 3	6	95.67	4.18		
glucose CSF/serum	CIS	11	0.76	0.03	14.95	<0.001
	RRMS cluster 1	21	0.71	0.02		
	RRMS cluster 2	8	0.77	0.07		
	RRMS cluster 3	6	1.1	0.07		
IgG CSF	CIS	11	3.47	0.39	2.450	0.076
	RRMS cluster 1	21	5.51	0.53		
	RRMS cluster 2	10	5.71	0.99		
	RRMS cluster 3	6	4.32	0.55		
IgG CSF/ IgG serum × 1000	CIS	11	3.47	0.37	3.523	0.023
	RRMS cluster 1	21	6.21	0.62		
	RRMS cluster 2	10	5.36	0.76		
	RRMS cluster 3	6	4.52	0.52		
IgG INDEX	CIS	11	0.64	0.04	2.321	0.088
	RRMS cluster 1	21	1.05	0.14		
	RRMS cluster 2	10	0.88	0.08		
	RRMS cluster 3	6	0.76	0.07		
brain T2W lesions **	CIS	10	21.2	3.27	0.339	0.797
	RRMS cluster 1	21	18.24	1.38		
	RRMS cluster 2	9	17.89	3.24		
	RRMS cluster 3	6	17	6.36		
brain Gd(+) lesions	CIS	11	0	0	1.682	0.185
	RRMS cluster 1	20	1	0.38		
	RRMS cluster 2	10	1.5	0.72		
	RRMS cluster 3	6	1	0.52		
spinal T2W lesions	CIS	10	0.6	0.16	2.069	0.120
	RRMS cluster 1	18	1. 8	0.45		
	RRMS cluster 2	9	2	0.53		
	RRMS cluster 3	6	2. 7	1.02		
spinal Gd(+) lesions	CIS	10	0	0	2.159	0.108
	RRMS cluster 1	18	0.28	0.16		
	RRMS cluster 2	9	1	0.47		
	RRMS cluster 3	6	0.5	0.5		
infratentorial and spinal T2 lesions	CIS	9	0.56	0.18	2.587	0.068
	RRMS cluster 1	18	1.78	0.45		
	RRMS cluster 2	8	1.88	0.58		
	RRMS cluster 3	6	3	0.86		

HCL: Hierarchical Clustering; CIS: Clinically Isolated Syndrome; RRMS: Relapsing-Remitting Multiple Sclerosis; S.E.: Standard Error; EDSS: Expanded Disability Status Scale; ALP: Alkaline Phospatase; TSH: thyroid stimulating hormone; ESR: Erythrocyte Sedimentation Rate; CSF: Cerebrospinal fluid; IgG: Immunoglobulin G; Q IgG: quotient of immunoglobulin G; T2W: T2-weighted; Gd(+): gadolinium-enhancing. * comparison: One-Way Analysis of Variance between Groups. Of routine serum laboratory parameters, only variables with significant and/or a tendency for significant difference are presented. ** data derived from VolBrain^TM^ lesion analysis.

**Table 4:** Metabolites implicated in the differentiation between the sub-groups (clusters) of patients with Relapsing-Remitting Multiple Sclerosis, derived from Hierarchical Clustering analysis.

Compounds	RRMS Cluster 3 vs. 1			RRMS Cluster 3 vs. 2		
	*t*-Tests	AUC	Log2 FC	*t*-Tests	AUC	Log2 FC
Acetylcarnitine	0.0064251	0.80159	−0.7736	0.015338	0.88333	−0.91828
Alanine	6.007 × 10^−5^	0.94444	−0.55022	0.0006984	0.96667	−0.50571
Arginine	0.0089912	0.75397	−0.482	0.0030095	0.91667	−0.75006
Asparagine	0.0003576	0.85714	−0.60183	0.0048269	0.83333	−0.45923
Aspartic_acid	N/A	N/A	N/A	0.0034009	0.86667	0.8524
Choline	N/A	N/A	N/A	0.0001562	1	0.4611
Creatinine	0.039939	0.76984	0.34011	N/A	N/A	N/A
Cystine	0.0044975	0.95238	1.613	0.017129	0.86667	1.0331
Glucose	0.0002488	0.84921	−0.51357	0.0024325	0.9	−0.71125
Glutamic_acid	N/A	N/A	N/A	0.0060747	0.8	0.61686
Glutamine	0.0006285	0.7619	−0.50125	0.018247	0.73333	−0.45563
Hydroxyisobutyric	2.155 × 10^−6^	0.96032	−1.3397	0.0001444	0.96667	−1.382
Lactic_acid	0.0076353	0.80952	−0.57308	N/A	N/A	N/A
Mannose	0.0005278	0.80952	−0.44569	0.0016796	0.91667	−0.69713
Methionine	3.259 × 10^−6^	0.96032	−1.1363	0.0007746	0.96667	−0.83975
MethylHippuric_acid	0.038958	0.77778	0.65068	N/A	N/A	N/A
Nicotinamide	0.046064	0.78571	0.88572	0.001909	0.96667	2,0052
Phenylalanine	3.324× 10^−7^	0.97619	−0.46776	0.0018768	0.9	−0.23945
Pyruvic_acid	6.703 × 10^−6^	0.96032	−1.154	0.0007994	0.95	−1.4189
Serine	0.0082229	0.81746	−0.29768	N/A	N/A	N/A
Taurine	N/A	N/A	N/A	0.0077124	0.86667	0.879
Theobromine	N/A	N/A	N/A	0.024328	0.91667	2.5868
Threonine	0.042464	0.74603	−0.40446	N/A	N/A	N/A
Uridine	0.034079	0.81746	0.3817	0.0006818	0.95	0.60553
Xanthine	N/A	N/A	N/A	0.0074553	0.91667	0.75873

Only statistically significant metabolites together with the results from univariate analysis including *t*-test, fold change analysis and area under the curve are summarized. RRMS: Relapsing-Remitting Multiple Sclerosis; Log2 FC: logarithmic 2-fold change; AUC: area under the curve; N/A: non-available.

## Data Availability

The datasets generated during and/or analysed during the current study are available from the corresponding author on reasonable request.

## Supplementary Materials

The following supporting information can be downloaded at: https://www.mdpi.com/article/10.3390/ijms232314578/s1.
