# Supplementary material for: Blood Metabolomics May Discriminate a Sub-Group of Patients with First Demyelinating Episode in the Context of RRMS with Increased Disability and MRI Characteristics Indicative of Poor Prognosis
Source: Int J Mol Sci. 2022 Nov 23;23(23):14578. doi: 10.3390/ijms232314578 (PMC9735785; doi:10.3390/ijms232314578)
Supplement: Supplementary file 1 [file ijms-23-14578-s001.zip › ijms-1973033-supplementary.pdf]

Supplementary Materials and Methods

1.1. Study Population

All participants were recruited at the Multiple Sclerosis (MS) Center of the 2nd University Neurological Clinic of A.U.TH. in the University General Hospital of Thessaloniki AHEPA, following written informed consent. The Study received approval of the Research Ethics and Conduct Committee of the A.U.TH. (AEDE AUTH) [Approval Nr. 112730/2021].

Inclusion criteria were age between 18 and 55 years, as well as fulfilment of one of the following: 1) patients with first demyelinating episode that did not meet criteria for the diagnosis of RRMS [1], more specifically, patients with objective neurological signs indicative of demyelination, continuous for at least 24 hours in the absence of dissemination in space (DIS) and/or dissemination in time (DIT) as defined in [1] (CIS Group), 2) patients with the first demyelinating episode that fulfilled diagnostic criteria for RRMS [1] (RRMS Group), and 3) healthy controls hospitalized and under evaluation for symptoms unrelated to an autoimmune and/or inflammatory neurological disease (e.g. headache) and/or healthy controls in the absence of hospitalization (Control group). Exclusion criteria were evidence of other/systemic inflammatory, autoimmune or neurodegenerative neurological disease, exposure to immunosuppressive or immunomodulatory agents, diabetes mellitus and/or other systemic disease that imposes dietary restrictions, use of corticosteroids 2 months prior to the study inclusion and clinical or laboratory evidence of current infectious disease.

1.2. Study procedures

<u>Data collection</u>: Demographics, personal health history, comorbidities, family health history, concomitant medication, history of the present disease, body mass index (BMI) and dietary parameters were collected for all participants at recruitment. Routine laboratory evaluation included haematological and biochemistry panel, thyroid function, B12, folate and Vit D assessment. All participants with CIS and/or RRMS were evaluated for vitamin D, B, Folate and TSH in the context of hospitalization and diagnostic evaluation for a demyelinating episode. For control participants that were not hospitalized, no indication for such evaluation was present, therefore not all control participants were evaluated for these parameters and these parameters were not taken into account for control participants. Of note, control participants did not present thyroid disease, did not report vitamin insufficiency in their personal history and did not receive vitamin supplementation upon sampling and at least six months prior to

inclusion in the present study. For patients with a first demyelinating episode full serum panel for autoimmune disease was evaluated, as well as evaluation of CSF, following lumbar puncture, for the presence of cells, biochemistry, IgG and albumin index and the presence of oligoclonal bands (OCBs). In addition for these patients, disability status by the use of Expanded Disability Status Scale (EDSS) was recorded [2]. These procedures were conducted in the frame of clinical diagnostic evaluation.

MRI evaluation: Brain and cervical spinal cord MRI with gadolinium administration was performed in the frame of routine clinical setting in various facilities. However, all MRI studies were evaluated by the treating Neurologists of the Center, all of whom have at least 5-year experience in treating patients with MS. Moreover, one treating Neurologist independently evaluated all MRI studies for reasons of internal consistency and internal quality control. Volumetry and lesion load analysis was conducted by the use of the VolBrain™ platform [3], following anonymization.

Sampling, serum collection and preparation for metabolomics analysis**:** All samples were collected under morning fasting conditions and diagnostic laboratory analyses were conducted prior to steroid administration. For metabolomics, whole peripheral blood was harvested and centrifuged for serum collection. Serum samples were stored at -80°C. For sample preparation for metabolomics analysis, samples were left to that at room temperature. After thawing, samples were vortex-mixed and an aliquot of 50 μL was diluted with 150 μL of acetonitrile (ACN) for protein precipitation. The resulting samples were vortexed vigorously for 1 min followed by centrifugation for 10 min at 15200g. Supernatants (200 μL) were transferred to LC-vials for analysis. A pooled sample (Quality Control Sample, QC) was prepared as a representative sample by mixing equal volumes of each test sample. The QC sample was treated as the test sample and was used to test the suitability and stability of the analytical system [4, 5] (**Supplementary Figure S1**). LC/MS reagents and solvents were of LC-MS grade; protocols and analysis were based on method previously developed by the authors [6]**.**

1.3. LC/MS reagents, protocols and analysis for metabolomics

Reagents and materials: LC/MS-grade acetonitrile (ACN) and ammonium formate (AF) were obtained from Merck (Darmstadt, Germany) and Sigma Aldrich (Gillingham, Dorset, UK) respectively. Water (18.2 M cm) was purified in Milli-Q device, Millipore Purification System (Merck Darmstadt, Germany).

Targeted Analysis: Targeted analysis of polar fraction was performed using a validated hydrophilic interaction liquid chromatography-tandem MS (HILIC-LC-

MS/MS) method monitoring ca 100 endogenous metabolites [6]. Chromatographic separations were carried out on an Acquity UPLC System (Waters Corporation, Milford, USA) using an Acquity UPLC BEH Amide column (Waters Ltd., Elstree, UK). MS detection was performed on a Xevo TQD mass spectrometer (Waters Corporation, Millford, USA) operating in both positive and negative ESI modes. A binary mobile phase system was used consisted of of A) acetonitrile-water, 95:5 (v/v) and B) acetonitrile-water, 30:70 (v/v), both containing 10 mM ammonium formate. The samples were analyzed in a randomized order and a QC sample was analyzed every after 10 samples within the analytical batch in order to assess analytical system stability.

Data processing and statistical analysis**:** Targeted analysis data were analyzed with MassLynx® and TargetLynx® (v4.1) (Waters, Milford, MA, USA). Raw data were normalized using the median fold change normalization algorithm. Normalised data were studied further in order to find any metabolic differences between the case-control groups by both multivariate and univariate statistical analysis tools. The SIMCA package (version 13.0.2.; Umetrics, Sweden) was used for multivariate statistical analysis and biomarker assessment via VIP plots (Variable Importance for the Projection), loading plots, S-plots, p(corr), and hotelling's lines. Principal Components Analysis (PCA) and Orthogonal Projection to Latent Structures Discriminant Analysis (OPLS-DA) were performed to assess data in a multivariate setting. Model validation was evaluated using permutation plots and CV-ANOVA value. The OPLS-DA models were validated by CA-ANOVA and R2Y, and Q2Y values. Within results we present results from models the cross- verification of the model’s validity. Univariate analysis using two-tailed t-test, with unequal variance algorithm (a threshold of p-value was set at 0.05) was performed to check the impact of each metabolite on the tested hypothesis. Intergroup variance was taken into account. There were no missing values. Network and path analysis was performed with MetPA (http://www.metaboanalyst.ca) a web-based tool dedicated to the analysis and visualization of metabolomic data within the biological context of metabolic pathways. Area Under the Curve was calculated using R programming language by measuring sensitivity and specificity.

For the analysis of continuous clinical data One-Way Analysis of Variance (ANOVA) or Kruskal-Wallis tests were conducted for the comparison of means, following normality test via the Kolmogorov-Smirnoff normality test. Bonferroni’s Post-Hoc comparisons were conducted, where applicable. For the analysis of categorical data Pearson’s chi-square was conducted. Statistical significance was set at $\alpha=0.05$.

The analysis was conducted by the use of SPSS for Windows (IBM® SPSS® Statistics 27.0).

Supplementary Figure S1.

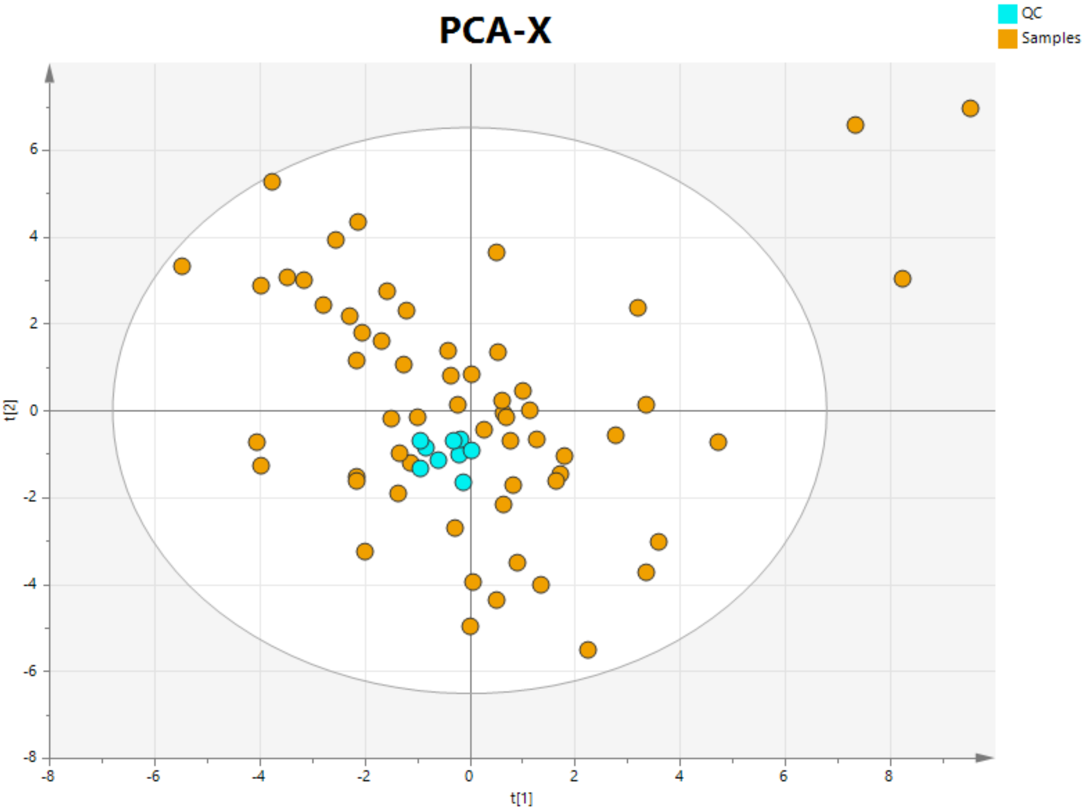

Supplementary Figure S1. PCA-X Score plot showing the tight cluster of QC (blue dots) samples.

Supplementary Table S1

Supplementary Table S1. Forty-six metabolites detected in blood samples including amines, amino acids, organic acids, carbohydrates, purines and other polar metabolites.

Glycine	Methionine	Valine	Cystine	Glutamine
Trimethylamine_n_oxide	Xanthine	Threonine	Adenosine	Lysine
Monoisoamylamine	Phenylalanine	Nicotinamide	Inosine	Glutamic_acid
Alanine	Arginine	Taurine	Pyruvic_acid	Mannose
Choline	Cotinine	Pyroglutamic	Lactic_acid	MethylHippuric_acid
Serine	Theobromine	isoleucine	Hydroxyisobutyric	Uridine
Cytosine	Tyrosine	Creatine	Hydroxyisovalericacid	Glucose
Kreatinine	Caffeine	Asparagine	Adipic_acid	Hypoxanthine
Proline	Acetylcarnitine	Aspartic_acid	Hippuric_acid	Betaine
Tryptophan				

Supplementary Table S2.

Supplementary Table S2. Magnetic Raisonance Imaging volumetry and lesion analysis parameters for patients with Clinically Isolated Syndrome and Relapsing – Remitting Multiple Sclerosis stratified into three clusters (1-3) derived from Hierarchical Cluster Analysis on the basis of serum metabolomic profile

Parameter / HCL Group		N	Mean	S.E. of Mean	F*	p*
Tissue WM cm3 Volume	CIS	11	472.50	30.18	0.400	0.753
	RRMS cluster 1	19	454.47	21.74		
	RRMS cluster 2	9	491.82	31.24		
	RRMS cluster 3	6	484.15	21.38		
Tissue WM % Volume	CIS	11	34.01	1.40	1.404	0.255
	RRMS cluster 1	19	32.81	1.23		
	RRMS cluster 2	9	37.56	2.72		
	RRMS cluster 3	6	34.96	1.90		
Tissue GM cm3 Volume	CIS	11	757.39	32.40	1.057	0.378
	RRMS cluster 1	19	734.76	32.19		
	RRMS cluster 2	9	668.06	43.70		
	RRMS cluster 3	6	770.38	50.48		
Tissue GM % Volume	CIS	11	54.96	1.77	0.944	0.428
	RRMS cluster 1	19	52.97	1.52		
	RRMS cluster 2	9	50.46	2.65		
	RRMS cluster 3	6	55.19	2.55		
Tissue CSF cm3 Volume	CIS	11	153.24	15.71	1.297	0.288
	RRMS cluster 1	19	192.09	21.48		
	RRMS cluster 2	9	158.48	11.67		
	RRMS cluster 3	6	136.96	23.17		
Tissue CSF % Volume	CIS	11	11.02	0.97	1.419	0.251
	RRMS cluster 1	19	14.23	1.69		
	RRMS cluster 2	9	11.98	0.78		
	RRMS cluster 3	6	9.86	1.60		
Tissue Brain cm3 Volume	CIS	11	1229.90	47.32	0.593	0.623
	RRMS cluster 1	19	1189.23	43.81		
	RRMS cluster 2	9	1159.89	30.29		
	RRMS cluster 3	6	1254.52	40.82		
Tissue Brain % Volume	CIS	11	88.98	0.97	1.421	0.250
	RRMS cluster 1	19	85.77	1.69		
	RRMS cluster 2	9	88.02	0.78		

	RRMS cluster 3	6	90.15	1.60		
Tissue IC cm3 Volume	CIS	11	1383.14	53.64	0.523	0.669
	RRMS cluster 1	19	1381.33	32.90		
	RRMS cluster 2	9	1318.37	34.95		
	RRMS cluster 3	6	1391.48	35.28		
Cerebrum Total cm3 Volume	CIS	11	1068.91	42.87	0.617	0.608
	RRMS cluster 1	19	1024.82	38.23		
	RRMS cluster 2	9	1002.81	29.14		
	RRMS cluster 3	6	1080.96	34.02		
Cerebrum Total % Volume	CIS	11	77.29	0.87	1.546	0.217
	RRMS cluster 1	19	73.94	1.51		
	RRMS cluster 2	9	76.07	0.94		
	RRMS cluster 3	6	77.68	1.31		
Cerebrum T GM cm3 Volume	CIS	11	643.80	27.36	1.124	0.351
	RRMS cluster 1	19	620.65	28.23		
	RRMS cluster 2	9	564.91	36.65		
	RRMS cluster 3	6	656.17	41.63		
Cerebrum T GM % Volume	CIS	11	46.70	1.42	1.118	0.353
	RRMS cluster 1	19	44.72	1.35		
	RRMS cluster 2	9	42.65	2.16		
	RRMS cluster 3	6	47.02	2.08		
Cerebrum T WM cm3 Volume	CIS	11	425.11	26.09	0.420	0.740
	RRMS cluster 1	19	404.17	19.00		
	RRMS cluster 2	9	437.90	26.71		
	RRMS cluster 3	6	424.78	18.86		
Cerebrum T WM % Volume	CIS	11	30.59	1.12	1.458	0.240
	RRMS cluster 1	19	29.20	1.10		
	RRMS cluster 2	9	33.42	2.33		
	RRMS cluster 3	6	30.66	1.64		
Cerebelum Total cm3 Volume	CIS	11	138.18	4.56	0.790	0.506
	RRMS cluster 1	19	140.99	5.25		
	RRMS cluster 2	9	134.85	3.32		
	RRMS cluster 3	6	149.16	6.48		
Cerebelum Total % Volume	CIS	11	10.04	0.23	0.752	0.527
	RRMS cluster 1	19	10.17	0.23		
	RRMS cluster 2	9	10.27	0.31		
	RRMS cluster 3	6	10.71	0.29		
Cerebelum T GM cm3 Volume	CIS	11	103.15	4.74	0.512	0.676
	RRMS cluster 1	19	104.76	4.48		
	RRMS cluster 2	9	95.39	7.35		
	RRMS cluster 3	6	104.74	8.98		
	CIS	11	7.52	0.34	0.176	0.912

Cerebelum T GM % Volume	RRMS cluster 1	19	7.59	0.26		
	RRMS cluster 2	9	7.22	0.52		
	RRMS cluster 3	6	7.49	0.51		
Cerebelum T WM cm3 Volume	CIS	11	34.85	5.02	0.749	0.529
	RRMS cluster 1	19	36.25	2.61		
	RRMS cluster 2	9	39.46	5.45		
	RRMS cluster 3	6	44.42	4.35		
Cerebelum T WM % Volume	CIS	11	2.52	0.33	1.138	0.345
	RRMS cluster 1	19	2.59	0.15		
	RRMS cluster 2	9	3.05	0.45		
	RRMS cluster 3	6	3.22	0.34		
Brainstem cm3 Volume	CIS	11	22.84	1.15	0.364	0.779
	RRMS cluster 1	19	23.45	1.19		
	RRMS cluster 2	9	22.25	1.16		
	RRMS cluster 3	6	24.46	1.17		
Brainstem % Volume	CIS	11	1.65	0.06	0.286	0.835
	RRMS cluster 1	19	1.69	0.06		
	RRMS cluster 2	9	1.68	0.07		
	RRMS cluster 3	6	1.76	0.08		
Lateral ventricles Total cm3 Volume	CIS	11	9.72	2.56	0.613	0.610
	RRMS cluster 1	19	13.12	3.02		
	RRMS cluster 2	9	13.49	2.64		
	RRMS cluster 3	6	7.85	1.91		
Lateral ventricles Total % Volume	CIS	11	0.68	0.16	0.773	0.516
	RRMS cluster 1	19	0.95	0.22		
	RRMS cluster 2	9	1.04	0.22		
	RRMS cluster 3	6	0.57	0.14		
Caudate Total cm3 Volume	CIS	11	6.32	0.37	0.452	0.717
	RRMS cluster 1	19	6.63	0.23		
	RRMS cluster 2	9	6.94	0.41		
	RRMS cluster 3	6	6.54	0.68		
Caudate Total % Volume	CIS	11	0.46	0.03	1.247	0.305
	RRMS cluster 1	19	0.48	0.02		
	RRMS cluster 2	9	0.53	0.04		
	RRMS cluster 3	6	0.47	0.04		
Putamen Total cm3 Volume	CIS	11	8.14	0.61	1.076	0.370
	RRMS cluster 1	19	7.33	0.28		
	RRMS cluster 2	9	7.26	0.50		
	RRMS cluster 3	6	6.89	0.63		
Putamen Total % Volume	CIS	11	0.59	0.04	1.552	0.216
	RRMS cluster 1	19	0.53	0.02		
	RRMS cluster 2	9	0.55	0.03		

	RRMS cluster 3	6	0.49	0.03		
Thalamus Total cm3 Volume	CIS	11	9.53	0.52	0.899	0.450
	RRMS cluster 1	19	8.48	0.33		
	RRMS cluster 2	9	9.01	0.69		
	RRMS cluster 3	6	9.16	0.88		
Thalamus Total % Volume	CIS	11	0.69	0.04	1.191	0.325
	RRMS cluster 1	19	0.62	0.03		
	RRMS cluster 2	9	0.68	0.04		
	RRMS cluster 3	6	0.66	0.05		
Globus Pallidus Total cm3 Volume	CIS	11	1.89	0.16	0.961	0.420
	RRMS cluster 1	19	1.77	0.13		
	RRMS cluster 2	9	1.96	0.18		
	RRMS cluster 3	6	1.49	0.30		
Globus Pallidus Total % Volume	CIS	11	0.14	0.01	1.565	0.212
	RRMS cluster 1	19	0.13	0.01		
	RRMS cluster 2	9	0.15	0.01		
	RRMS cluster 3	6	0.11	0.02		
Hipocampus Total cm3 Volume	CIS	11	7.33	0.69	0.097	0.961
	RRMS cluster 1	19	7.66	0.31		
	RRMS cluster 2	9	7.65	0.69		
	RRMS cluster 3	6	7.63	0.52		
Hippocampus Total % Volume	CIS	11	0.53	0.05	0.259	0.854
	RRMS cluster 1	19	0.56	0.02		
	RRMS cluster 2	9	0.58	0.05		
	RRMS cluster 3	6	0.55	0.03		
Amygdala Total cm3 Volume	CIS	11	1.54	0.15	1.208	0.319
	RRMS cluster 1	19	1.38	0.11		
	RRMS cluster 2	9	1.24	0.18		
	RRMS cluster 3	6	1.08	0.20		
Amygdala Total % Volume	CIS	11	0.11	0.01	1.130	0.348
	RRMS cluster 1	19	0.10	0.01		
	RRMS cluster 2	9	0.10	0.01		
	RRMS cluster 3	6	0.08	0.01		
Accumbens Total cm3 Volume	CIS	11	0.46	0.05	0.178	0.911
	RRMS cluster 1	19	0.45	0.03		
	RRMS cluster 2	9	0.45	0.10		
	RRMS cluster 3	6	0.40	0.08		
Accumbens Total % Volume	CIS	11	0.03	0.00	0.079	0.971
	RRMS cluster 1	19	0.03	0.00		
	RRMS cluster 2	9	0.03	0.01		
	RRMS cluster 3	6	0.03	0.01		
Scale factor	CIS	5	0.75	0.05	0.065	0.978

	RRMS cluster 1	12	0.77	0.02		
	RRMS cluster 2	5	0.75	0.01		
	RRMS cluster 3	3	0.75	0.03		
ICV cm3	CIS	10	1363.09	55.37	0.549	0.651
	RRMS cluster 1	21	1388.20	32.92		
	RRMS cluster 2	9	1313.76	38.97		
	RRMS cluster 3	6	1360.18	42.51		
Total lesion count	CIS	10	21.20	3.27	0.339	0.797
	RRMS cluster 1	21	18.24	1.38		
	RRMS cluster 2	9	17.89	3.24		
	RRMS cluster 3	6	17.00	6.36		
Total lesion Volume (absolute) cm3	CIS	10	17.42	13.90	0.916	0.442
	RRMS cluster 1	21	6.16	1.74		
	RRMS cluster 2	9	2.89	1.05		
	RRMS cluster 3	6	5.08	3.01		
Total lesion Volume (normalized) %	CIS	10	1.29	1.02	0.936	0.432
	RRMS cluster 1	21	0.45	0.13		
	RRMS cluster 2	9	0.22	0.08		
	RRMS cluster 3	6	0.35	0.20		
Total lesion burden	CIS	10	2.79	2.20	0.761	0.523
	RRMS cluster 1	21	1.24	0.43		
	RRMS cluster 2	9	0.54	0.17		
	RRMS cluster 3	6	0.83	0.49		
Periventricular lesion count	CIS	10	5.50	1.17	0.583	0.630
	RRMS cluster 1	21	6.33	0.81		
	RRMS cluster 2	9	6.67	0.75		
	RRMS cluster 3	6	4.67	0.99		
Periventricular lesion volumeume (absolute) cm3	CIS	10	15.78	13.94	0.808	0.497
	RRMS cluster 1	21	5.26	1.67		
	RRMS cluster 2	9	2.05	0.75		
	RRMS cluster 3	6	4.38	2.99		
Periventricular lesion volumeume (normalized) %	CIS	10	1.17	1.03	0.839	0.480
	RRMS cluster 1	21	0.38	0.13		
	RRMS cluster 2	9	0.15	0.05		
	RRMS cluster 3	6	0.30	0.20		
Periventricular lesion burden	CIS	10	2.56	2.20	0.708	0.552
	RRMS cluster 1	21	1.07	0.41		
	RRMS cluster 2	9	0.38	0.13		
	RRMS cluster 3	6	0.72	0.49		
Juxtacortical lesion count	CIS	10	7.30	1.16	0.320	0.811
	RRMS cluster 1	21	7.14	0.89		
	RRMS cluster 2	9	5.44	1.51		

	RRMS cluster 3	6	7.50	3.81		
Juxtacortical lesion Volume (absolute) cm3	CIS	10	1.14	0.34	1.340	0.274
	RRMS cluster 1	21	0.73	0.18		
	RRMS cluster 2	9	0.45	0.17		
	RRMS cluster 3	6	0.55	0.13		
Juxtacortical lesion Volume (normalized) %	CIS	10	0.08	0.02	1.326	0.279
	RRMS cluster 1	21	0.05	0.01		
	RRMS cluster 2	9	0.03	0.01		
	RRMS cluster 3	6	0.04	0.01		
Juxtacortical lesion burden	CIS	10	0.17	0.05	0.733	0.538
	RRMS cluster 1	21	0.14	0.03		
	RRMS cluster 2	9	0.09	0.03		
	RRMS cluster 3	6	0.09	0.02		
Deep white lesion count	CIS	10	8.40	3.84	0.788	0.507
	RRMS cluster 1	21	4.76	0.58		
	RRMS cluster 2	9	5.78	1.70		
	RRMS cluster 3	6	4.50	1.80		
Deep white lesion Volume (absolute) cm3	CIS	10	0.50	0.31	1.138	0.345
	RRMS cluster 1	21	0.16	0.04		
	RRMS cluster 2	9	0.39	0.23		
	RRMS cluster 3	6	0.11	0.04		
Deep white lesion Volume (normalized) %	CIS	10	0.04	0.02	1.330	0.277
	RRMS cluster 1	21	0.01	0.00		
	RRMS cluster 2	9	0.03	0.02		
	RRMS cluster 3	6	0.01	0.00		
Deep white lesion burden	CIS	10	0.06	0.03	1.099	0.360
	RRMS cluster 1	21	0.03	0.01		
	RRMS cluster 2	9	0.07	0.04		
	RRMS cluster 3	6	0.02	0.01		
**Infratentorial lesion count**	**CIS**	**10**	**0.00**	**0.00**	**6.087**	**0.002**
	**RRMS cluster 1**	**21**	**0.00**	**0.00**		
	**RRMS cluster 2**	**9**	**0.00**	**0.00**		
	**RRMS cluster 3**	**6**	**0.33**	**0.21**		
**Infratentorial lesion Volume (absolute) cm3**	**CIS**	**10**	**0.00**	**0.00**	**6.087**	**0.002**
	**RRMS cluster 1**	**21**	**0.00**	**0.00**		
	**RRMS cluster 2**	**9**	**0.00**	**0.00**		
	**RRMS cluster 3**	**6**	**0.04**	**0.03**		
**Infratentorial lesion Volume (normalized) %**	**CIS**	**10**	**0.00**	**0.00**	**6.087**	**0.002**
	**RRMS cluster 1**	**21**	**0.00**	**0.00**		
	**RRMS cluster 2**	**9**	**0.00**	**0.00**		
	**RRMS cluster 3**	**6**	**0.0033**	**0.002**		
	**CIS**	**10**	**0.00**	**0.00**	**6.087**	**0.002**

**Infratentorial lesion burden**	**RRMS cluster 1**	**21**	**0.00**	**0.00**		
	**RRMS cluster 2**	**9**	**0.00**	**0.00**		
	**RRMS cluster 3**	**6**	**0.01**	**0.004**		

HCL: Hierarchical Clustering; CIS: Clinically Isolated Syndrome; RRMS: Relapsing-Remitting Multiple Sclerosis; S.E.: Standard Error; WM: white matter; GM: grey matter; CSF: *Cerebrospinal fluid*; IC: intra-cerebral. * One-Way Analysis of Variance between Groups.

References

1. Thompson, A. J.; Banwell, B. L.; Barkhof, F.; Carroll, W. M.; Coetzee, T.; Comi, G.; Correale, J.; Fazekas, F.; Filippi, M.; Freedman, M. S.; Fujihara, K.; Galetta, S. L.; Hartung, H. P.; Kappos, L.; Lublin, F. D.; Marrie, R. A.; Miller, A. E.; Miller, D. H.; Montalban, X.; Mowry, E. M.; Sorensen, P. S.; Tintore, M.; Traboulsee, A. L.; Trojano, M.; Uitdehaag, B. M. J.; Vukusic, S.; Waubant, E.; Weinshenker, B. G.; Reingold, S. C.; Cohen, J. A., Diagnosis of multiple sclerosis: 2017 revisions of the McDonald criteria. *Lancet Neurol* **2018,** 17, (2), 162-173.
2. Kurtzke, J. F., Rating neurologic impairment in multiple sclerosis: an expanded disability status scale (EDSS). *Neurology* **1983,** 33, (11), 1444-52.
3. Manjon, J. V.; Coupe, P., volBrain: An Online MRI Brain Volumetry System. *Frontiers in neuroinformatics* **2016,** 10, 30.
4. Gika, H. G.; Theodoridis, G. A.; Wingate, J. E.; Wilson, I. D., Within-day reproducibility of an HPLC-MS-based method for metabonomic analysis: application to human urine. *Journal of proteome research* **2007,** 6, (8), 3291-303.
5. Gika, H. G.; Macpherson, E.; Theodoridis, G. A.; Wilson, I. D., Evaluation of the repeatability of ultra-performance liquid chromatography-TOF-MS for global metabolic profiling of human urine samples. *Journal of chromatography. B, Analytical technologies in the biomedical and life sciences* **2008,** 871, (2), 299-305.
6. Virgiliou, C.; Sampsonidis, I.; Gika, H. G.; Raikos, N.; Theodoridis, G. A., Development and validation of a HILIC-MS/MS multitargeted method for metabolomics applications. *Electrophoresis* **2015,** 36, (18), 2215-2225.
